# *Memórias do Instituto Oswaldo Cruz* celebrates 115 years of scientific publishing: what it needs to keep moving on…

**DOI:** 10.1590/0074-02760240003

**Published:** 2024-05-27

**Authors:** Adeilton Alves Brandão, Ana Carolina P Vicente

**Affiliations:** 1 Fundação Oswaldo Cruz-Fiocruz, Instituto Oswaldo Cruz, Rio de Janeiro, RJ, Brasil

The Federal Decree n. 1802, published in December 12th 1907,^1^ determined the creation of the “Instituto de Pathologia Experimental de Manguinhos”, now the “Instituto Oswaldo Cruz” and one of the current 20 research institutes of “Fundação Oswaldo Cruz - Fiocruz”. The same decree also determined the creation of a journal to disseminate the research work of the ‘Instituto’, stating in its 9th paragraph: “The research work carried out at the ‘Instituto de Manguinhos’ will be published as *Memorias* soon after confirming the experiments.” The first printed edition of the journal *Memórias do Instituto Oswaldo Cruz* appeared in April 1909, with the publication of eight articles exclusively from the “Instituto de Manguinhos“. Articles were published in dual language, Portuguese and either German or French. That was the beginning of a scientific journal that has demonstrated resilience and adaptation to the huge political, economic and social transformations that Brazil has suffered since the end of the 19th century. It has survived to become a centennial journal that keeps publishing research work in human infectious diseases, their agents and vectors in Latin America. Of course, *Memorias do IOC* is also a proud member of the exclusive and “highly selective” set of scientific journals in the world that has celebrated more than a century of publishing activity.

The centenary milestone notwithstanding, *Memórias do IOC* pathway has not been a cool journey to success and recognition. Following the turbulent period of two World Wars and the consequences for the biomedical science research, *Memórias do IOC* existence was severely threatened during the “hard years” of 1960 and 70’s decades, when intramural instability, administrative reorganisation and economic crisis under the military dictatorship in Brazil summed up to create a “storm” that caused an interruption of its publishing operation. The journal did not publish any article during the years of 1977-1979. That was an inflexion point in *Memórias do IOC* pathway, and to give a wide view of the journal till this decadency period, let us remember its early operational years taking as reference the publishing practice of those earlier decades.

The first point worth mentioning is the feature “Institutional Journal”, that is, a vehicle dedicated to publish solely the research work of researchers affiliated to the so-called “Instituto de Manguinhos.” No research work outside the “Instituto de Patologia Experimental de Manguinhos” (the second official name), renamed later “Instituto Oswaldo Cruz”, could be published by *Memórias do IOC*.^2^ In the beginning of 20th century some Research Institutes used to edit and publish its own and (almost) exclusive journal. The model was based on the practice of the Institute Pasteur, in France, that started publishing the journal Annales de l’Institute Pasteur in 1887.^3^ The initiative to sponsor a home journal was observed in other European research institute, for example, The Liverpool School of Tropical Medicine (LSTM), and the “Instituto de Higiene e Medicina Tropical” in Lisbon, Portugal. It is important to remind that the institutes of Tropical Medicine (and the respective scholarly societies) at the end of 19th century were to a large extent linked to the colonialist operation promoted by European countries. In addition to training in the “medicine of the tropics”, the technical staff of these Tropical Medicine institutes provided some scientific support to the success of the European colonialist venture in Africa and Southeast Asia.^4^

Towards the end of 20th century, journals published by these institutes and scholarly societies were rebranded, closed or outsourced to big publishing houses. For example, the Annals of Tropical Medicine and Parasitology, published by the LSTM was sold in 2002 (and later renamed as Pathogens and Global Health) to the publisher Maney Publishing (acquired by Taylor & Francis in 2015),^5^ ^,^ ^6^ and the old Annales de l’Institute Pasteur (currently known as Research in Microbiology) is published now by the Elsevier.^7^ The rebranding/outsourcing was, in part, a kind of alignment to the contemporaneous scientific publication practice, by which the scholars have transferred the job of publishing/disseminating articles from university/research institute/scholarly society to commercial (for-profit) publishers. Such a decision has had a tremendous impact in the way science is communicated to society, and a debate is ongoing in the world whether it was an appropriate decision given the potential conflict of interests that arises when publishers need to choose between publishing more articles and complying with academic rigour (let us not forget the appearance of “rent/money seeking” publishers a.k.a “predators publishers”). A note on the term “predatory publisher/journal”: although this term was coined to describe the deleterious activity some publishers/journals have on scientific publishing environment,^8^ it is not a proper term. Rigorously, predator/predatory is an ecological term with a precise meaning, and the predators in nature has a very positive role, maintaining the delicate equilibrium typical of natural ecosystems. Thus, to avoid confusion, a proper definition for the deleterious activity of these publishers is “rent seeking publishers” or “money-hunter” publishers. Whatever the word to properly name this practice, the truth is that they are gradually destroying the credibility of science!

The question we should ask considering the above mentioned facts is whether an institutional journal might have a place in the current publishing scenario. Taking into account the publishing model that *Memórias do IOC* practised at the dawn the 20th century the answer is clearly “no”. The *modus operand*i of science in those days was different for reasons such as: (i) there was no “clear” connection between scientific achievement and “national strategy” as recognised in the post-world War II period^9^ (although the “use” of science for both colonialist and military purpose was common in Europe); (ii) communication of scientific achievement was in the national languages (there was no ‘língua franca’ for science like the English language today); for instance, *Memórias do IOC* published the articles simultaneously in Portuguese and either French or German, what was a quite unusual and interesting editorial decision for a nascent journal in a country located in Southern hemisphere; (iii) In Brazil at that moment, science was not “institutionalised”, *i. e.*, there was no governmental agency to neither support science nor to develop a national strategy. The institutions that help giving a “shape” to Brazilian science, CNPq and CAPES, were both created several decades later, in 1951.

The Brazilian Republic was born in November 15th, 1889, and soon was immersed in a political/economic crisis (this is almost a constant in our republican history). Science, was then, a very exceptional activity in our country, and for this reason, the first publishing period of *Memórias do IOC* (the original institutional phase) is a tale of a “surviving journal”, going arms in arms with its “sponsor organisation” (Instituto Oswaldo Cruz/Fiocruz) in the “long and winding road” of proposing solutions to the public health challenges in Brazil.

In contrast to institutional journals that changed their publishing practices (and also the “appearance”) to become relevant in the post-world War II, *Memórias do IOC* has more or less kept its original features. Officially, *Memórias do IOC* was not an exclusively institutional journal in the years following 1960,^10^ but most of the articles was authored by researchers from Instituto Oswaldo Cruz. Thus, no major change in publishing practice. The scientific world was changing rapidly, and the golden days of institutional journals before long would turn out to be reminiscences. Now enter the publishing industry and its enormous influence and economic power, in a level strong enough to interrupt a prepublication article sharing experiment conducted by the National Institutes of Health (NIH) in 1961-1967 (this was the first attempt to implement what we call today “preprint article”).^11^

In this new world, the scientific article mutates from a standardised kind of letter conveying research results to a “product” that should be counted and measured as goals to be met in order to a researcher get funded and promoted. It still conveys research results in a standard format but now the article content is not the first element to be seen by the readers. Rather is the journal where it has been published the most important element of this equation. As a product indicating performance, quantity first matters followed by journal quality/impact.

The scientometrics comes of age, and for journals like *Memórias do IOC*, which are still seen as ‘institutional’ one publishing local/regional research results, and located in scientific peripherally countries, was a kind of nightmare. The “best” articles are not sent to these journals as first option, creating a “vicious” publishing cycle: neither relevant nor novel research reports are published by these journals, and by consequence they will not gather citations (another important metrics of 20th century science) and have its “prestige” reduced at each evaluation cycle (usually 2-5 years). In other words, if a journal only publishes non relevant research work, it will be a non-relevant journal. How to overcome this self-reproduced barrier if researchers are reluctant to send their “best” papers to a “non-prestigious” journal? The answer to this question is open, solutions have been proposed but none of them says how to get rid of the powerful incentives that forces (or stimulates) researchers towards the “prestige” journal.^12^ ^,^ ^13^ Rephrasing this question: how to become a “prestigious” journal? Or more fundamentally, what is “prestige” for science? We will let these questions without answer, we don’t have them yet. And for what is our concern here, over the last few decades *Memórias do IOC* has been tiered among the most relevant scientific journals in the fields of Tropical Medicine and Parasitology.

There is a last question, and it concerns the next 100 years if this journal is supposed to get there: what changes should *Memórias do IOC* promote to keep its publishing activity and also be perceived as a “relevant” scientific journal? For the present, the doom day appears not so close, as *Memórias do IOC* centennial history might count as a protective layer, displaying “credibility” and “tradition”. Under certain circumstances, this is equivalent to an asset so that some people may ask for a “seal” to assure quality or prestige that will stamp the research work. In a scenario where the scientific publishing activity will be carried out mainly by “publishing platforms” (*e. g.*, immediate publication, real time peer review, and transparency of all procedures), the remaining role for a journal is... to recommend/endorse/impulse articles that have already been evaluated/validated by the research community using such platforms. How should this “recommendation” work? Should it be really relevant to the research community or simply a strategy to extend the journal’s life? It all depends on the above mentioned keyword: incentive! If the interests of the research community is aligned to the journal needs for survival through a “proper set of incentives”, *Mem*ó*rias do IOC* will have the breathe for another one hundred years celebration!

That is what we all hope and work for in the near future.
